# Evaluation of Host Defense Peptide (CaD23)-Antibiotic Interaction and Mechanism of Action: Insights From Experimental and Molecular Dynamics Simulations Studies

**DOI:** 10.3389/fphar.2021.731499

**Published:** 2021-10-07

**Authors:** Darren Shu Jeng Ting, Jianguo Li, Chandra S. Verma, Eunice T. L. Goh, Mario Nubile, Leonardo Mastropasqua, Dalia G. Said, Roger W. Beuerman, Rajamani Lakshminarayanan, Imran Mohammed, Harminder S. Dua

**Affiliations:** ^1^ Academic Ophthalmology, Division of Clinical Neuroscience, School of Medicine, University of Nottingham, Nottingham, United Kingdom; ^2^ Department of Ophthalmology, Queen’s Medical Centre, Nottingham, United Kingdom; ^3^ Anti-Infectives Research Group, Singapore Eye Research Institute, Singapore, Singapore; ^4^ Bioinformatics Institute (A*Star), Singapore, Singapore; ^5^ School of Biological Sciences, Nanyang Technological University, Singapore, Singapore; ^6^ Department of Biological Sciences, National University of Singapore, Singapore, Singapore; ^7^ Ophthalmic Clinic, University “G d’Annunzio” of Chieti-Pescara, Chieti, Italy

**Keywords:** antimicrobial peptide (AMP), cathelicidin (LL37), computational simulation, defensin, drug design, host defense (antimicrobial) peptides, molecular dynamics simulations, antibiotic

## Abstract

**Background/Aim:** Host defense peptides (HDPs) have the potential to provide a novel solution to antimicrobial resistance (AMR) in view of their unique and broad-spectrum antimicrobial activities. We had recently developed a novel hybrid HDP based on LL-37 and human beta-defensin-2, named CaD23, which was shown to exhibit good *in vivo* antimicrobial efficacy against *Staphylococcus aureus* in a bacterial keratitis murine model. This study aimed to examine the potential CaD23-antibiotic synergism and the secondary structure and underlying mechanism of action of CaD23.

**Methods:** Peptide-antibiotic interaction was evaluated against *S. aureus*, methicillin-resistant *S. aureus* (MRSA), and *Pseudomonas aeruginosa* using established checkerboard and time-kill assays. Fractional inhibitory concentration index (FICI) was calculated and interpreted as synergistic (FIC<0.5), additive (FIC between 0.5–1.0), indifferent (FIC between >1.0 and ≤4), or antagonistic (FIC>4). SYTOX green uptake assay was performed to determine the membrane-permeabilising action of CaD23. Molecular dynamics (MD) simulations were performed to evaluate the interaction of CaD23 with bacterial and mammalian mimetic membranes. Circular dichroism (CD) spectroscopy was also performed to examine the secondary structures of CaD23.

**Results:** CaD23-amikacin and CaD23-levofloxacin combination treatment exhibited a strong additive effect against *S. aureus* SH1000 (FICI = 0.60–0.69) and MRSA43300 (FICI = 0.56–0.60) but an indifferent effect against *P. aeruginosa* (FIC = 1.03–1.15). CaD23 (at 25 μg/ml; 2xMIC) completely killed *S. aureus* within 30 min. When used at sub-MIC concentration (3.1 μg/ml; 0.25xMIC), it was able to expedite the antimicrobial action of amikacin against *S. aureus* by 50%. The rapid antimicrobial action of CaD23 was attributed to the underlying membrane-permeabilising mechanism of action, evidenced by the SYTOX green uptake assay and MD simulations studies. MD simulations revealed that cationicity, alpha-helicity, amphiphilicity and hydrophobicity (related to the Trp residue at C-terminal) play important roles in the antimicrobial action of CaD23. The secondary structures of CaD23 observed in MD simulations were validated by CD spectroscopy.

**Conclusion:** CaD23 is a novel alpha-helical, membrane-active synthetic HDP that can enhance and expedite the antimicrobial action of antibiotics against Gram-positive bacteria when used in combination. MD simulations serves as a powerful tool in revealing the peptide secondary structure, dissecting the mechanism of action, and guiding the design and optimisation of HDPs.

## Introduction

Antimicrobial resistance (AMR) is currently one of the major global health threats. (Prestinaci et al., 2015; Ventola, 2015) By 2050, it is estimated to cause 10 million deaths and cost the global economy up to 100 trillion USD if the issue remains untackled. (O’Neill, 2016) In addition, non-systemic infections, including ocular and skin infections, are being increasingly affected by drug-resistant pathogens, which usually result in poor prognosis (Ventola, 2015; Pulido-Cejudo et al., 2017; Ting et al., 2021a). In view of the colossal impact on global health and economy, various initiatives and strategies have been proposed and implemented to tackle AMR. These include establishment of antimicrobial stewardship to monitor the use of antimicrobial agents and the rise of AMR, development of new drugs and vaccines, drug repurposing, and incentivising pharmaceutical companies for investing in antimicrobial drug development. (Ventola, 2015)

Infectious keratitis (IK) represents the fifth leading cause of blindness globally. (Ting et al., 2021a) It can be caused by a wide range of organisms, including bacteria, fungi, viruses, and parasites, particularly Acanthamoeba. (Shah et al., 2011; Khor et al., 2018; Ting et al., 2018; Ting et al., 2019a; Green et al., 2019; Khoo et al., 2020; Ting et al., 2021b; Hoffman et al., 2021) Broad-spectrum topical antibiotic treatment is the current mainstay of treatment for IK, but the management is being challenged by the low culture yield, (Ting et al., 2021a; Ting et al., 2021b) polymicrobial infection, (Tu et al., 2009; Ting et al., 2019b; Khoo et al., 2020) and emerging AMR. (Hernandez-Camarena et al., 2015; Lalitha et al., 2017; Lin et al., 2019; Asbell et al., 2020) In addition, adjuvant procedures/surgeries such as therapeutic corneal cross-linking, (Ting et al., 2019c) amniotic membrane transplantation, (Ting et al., 2021c) and therapeutic/tectonic keratoplasty (Hossain et al., 2018; Ting et al., 2020a) are often required to manage uncontrolled infection and its complications, including corneal melting and perforation. All these issues highlight the need for new treatment for IK.

Host defense peptides (HDPs) have shown promise as a novel solution to AMR in view of their unique and broad-spectrum antimicrobial activities. (Mookherjee et al., 2020) These HDPs are usually highly cationic and amphiphilic, with ∼30–50% hydrophobicity. (Hancock and Lehrer, 1998; Mohammed et al., 2017; Haney et al., 2019) The cationic amino acid residues facilitate the binding of HDPs onto the anionic bacterial membrane (via electrostatic interactions), while the hydrophobic residues interact with the lipid tail region of the membrane, culminating in membrane disruption, leakage of cytoplasmic contents and subsequent cell death. (Hancock and Sahl, 2006) In addition to the direct antimicrobial activity, HDPs exhibit anti-biofilm, anti-tumour, immunomodulatory, chemotactic and wound-healing properties, offering a wide range of potential therapeutic applications. (Hancock et al., 2016; Mookherjee et al., 2020)

However, several barriers, including cytotoxicity to host cells and stability in the host/infective environment, have so far hindered the clinical translation of HDP-based antimicrobial therapy. (Ting et al., 2020b) To overcome these barriers, some research groups have explored the use of peptide-antibiotic combination therapy as a means to exploit the peptide-antibiotic synergistic effect for treating various types of infections. (Nuding et al., 2014; Lakshminarayanan et al., 2016; Pletzer et al., 2018; Kampshoff et al., 2019; Mohammed et al., 2019) This attractive antimicrobial strategy not only helps extend the lifespan and broaden the antibacterial spectrum of conventional antibiotics, but also reduces the dose-dependent toxicity associated with HDPs and antibiotics. (Mishra et al., 2017)

Recently, our group had demonstrated that CaD23, a hybrid derivative of human cathelicidin (LL-37) and human beta-defensin (HBD)-2, exhibited a more rapid *in vitro* antimicrobial action than conventional antibiotics such as amikacin, with no risk of AMR observed among the CaD23-treated bacteria. (Ting et al., 2021d) However, the mechanism of action has not been fully elucidated. In addition, while CaD23 exhibited reasonable *in vivo* efficacy at a concentration of 0.05% (500 μg/ml), the use of a higher concentration of CaD23 to achieve stronger antimicrobial effect was prohibited by the toxicity, as observed in the wound healing study. Therefore, to overcome this limitation, we aimed to examine the potential synergism/interaction between CaD23 and commonly used antibiotics for IK, including levofloxacin and amikacin. (Ting et al., 2021e) In addition, we aimed to determine the secondary structures and mechanism of action of CaD23 using a combination of experimental and molecular dynamics (MD) simulations studies.

## Materials and Methods

### Chemicals and Antibiotics

The synthetic hybrid peptide, CaD23 (sequence: KRIVQRIKDWLRKLCKKW), was commercially produced by Mimotopes (Mimotopes Pty. Ltd., Mulgrave Victoria, Australia) *via* traditional solid phase Fmoc synthesis method. CaD23 was purified by reverse-phase high performance liquid chromatography (RP-HPLC) to >95% purity and characterised by mass spectrometry (Supplementary Figure S1). In view of the hydrophobicity, CaD23 was first fully dissolved in 50 μL of dimethyl sulfoxide (DMSO) followed by dilution in sterile, de-ionised water to achieve a final concentration of 1 mg/ml peptide in 99.5% 0.5% v/v DMSO. Further dilution was performed for specific assays as required. All the assays described in this study were conducted in biological duplicate and in three independent experiments, with appropriate positive controls (PCs) and negative controls (NCs). Antibiotics, including levofloxacin and amikacin, were purchased from Sigma-Aldrich (Merck Life Science UK Ltd., Dorset, United Kingdom). The data were presented in mean ± standard deviation (SD).

### Types of Microorganisms Used

A range of Gram-positive and Gram-negative bacteria were used in this study. These included laboratory-strain methicillin-sensitive *Staphylococcus aureus* (SH1000 and ATCC SA29213), methicillin-resistant *S. aureus* (ATCC MRSA43300), *Pseudomonas aeruginosa* ATCC PA19660 (cytotoxic strain), and *P. aeruginosa* ATCC PA27853 (invasive strain). Both cytotoxic and invasive *P. aeruginosa* strains were used in the experiments as previous studies had demonstrated the difference in virulence. (Lee et al., 2003; Borkar et al., 2013)

### Determination of Antimicrobial Efficacy

*In vitro* antimicrobial efficacy of CaD23 and the antibiotics was determined using the established minimum inhibitory concentration (MIC) assay with broth microdilution method approved by the Clinical and Laboratory Standards Institute (CLSI). (Clinical and Laboratory Standards Institute (Clsi), 2019) Briefly, the microorganisms were cultured on Tryptone Soya Agar (TSA) and incubated overnight for 18–21 h at 37°C. Bacterial inoculums were subsequently prepared using the direct colony suspension method. (Clinical and Laboratory Standards Institute (Clsi), 2019) Three to five bacterial colonies were obtained from the agar plate and inoculated into an Eppendorf tube containing 1 ml of cation-adjusted Muller-Hinton broth (caMHB, Merck), consisting of 20–25 mg/L calcium ions (Ca^2+^) and 10–12.5 mg/L magnesium ions (Mg^2+^). The bacterial suspension was adjusted to achieve a turbidity equivalent to 0.1 OD_600_ or 0.5 MacFarland, containing ∼1.5 × 10^8^ colony-forming unit (CFU)/ml, which was then further diluted in 1:150 in caMHB to reach a final bacterial concentration of ∼1 × 10 (Ting et al., 2021b) colony forming units (CFU)/ml. Subsequently, 50 μL of 1 × 10 (Ting et al., 2021b) CFU/ml bacteria and 50 μL of treatment/controls were added into each well for the MIC assay. The MIC values, defined as the lowest concentration of the antimicrobial agent that prevented any visible growth of bacteria, were determined after 18–21 h of incubation at 37°C.

### Determination of the Peptide-antibiotic Interaction

The peptide-antibiotic interaction was determined using two methods, namely the checkerboard assay and the time-kill kinetics assay.

#### Checkerboard Assay

The peptide-antibiotic synergism was examined using the established checkerboard assay described in the previous study. (Mohammed et al., 2019) A 96-well polypropylene plate (Plate A) was used to prepare 8 replicate horizontal rows of CaD23 in twofold serial dilutions [from 400 μg/ml (first column) to 6.25 μg/ml (seventh column), and caMHB in the last (eighth) column; final volume of 25 μL per well]. Another 96-well polystyrene plate (Plate B) was used to prepare 8 replicate vertical columns of an antibiotic, either amikacin (an aminoglycoside) or levofloxacin (a fluoroquinolone), in twofold serial dilutions [from 20 μg/ml (first row) to 0.313 μg/ml (seventh row), and 0 μg/ml in the last (eighth) row; final volume of 30 μL per well]. Subsequently, 25 μL of antibiotic from each well of Plate B was transferred to the corresponding wells of Plate A (1:1 ratio of peptide and antibiotic). The bacterial suspension was prepared as above and 50 μL of 1 × 10 (Ting et al., 2021b) CFU/ml bacteria was added into each well (1:1 ratio of treatment and bacteria; final concentration of 5 × 10^5^ CFU/ml bacteria per well). The final concentration of CaD23 in each row was 100 μg/ml (first column) to 1.56 μg/ml (seventh column) and the final concentration of antibiotic in each column was 5 μg/ml (first row) to 0.078 μg/ml (seventh row). Growth control and sterility control were included in each experiment. The MIC was calculated as above after 18–21 h of incubation with treatment at 37°C.

The fractional inhibitory concentration index (FICI) is calculated using the formula: (MIC_CaD23(combined)_/MIC_CaD23(alone)_) + (MIC_antibiotic(combined)_/MIC_antibiotic(alone)_) and was interpreted as synergistic (FICI <0.5), additive (FICI between 0.5–1.0), indifferent (FICI between >1.0 and ≤4), or antagonistic (FICI >4).

#### Time-Kill Kinetics Assay

Time-kill kinetics assay was performed to determine the time and concentration-dependent antimicrobial activity of CaD23 and amikacin against SH1000. The bacterial suspension (with a concentration of 1 × 10^6^ CFU/ml) was prepared using the similar method as described in the MIC assay. 50 μL of bacteria was then incubated with 50 μL of respective treatment, consisting of either CaD23 alone, amikacin alone, or combined CaD23-amikacin. Bacterial suspension incubated with sterile de-ionised water (dH_2_O) in 1:1 ratio was used as the growth control. At 0, 15, 30 min, 1 h, 2 h, 4 h, and 24 h, 10 μL of the treatment/bacteria mixture was removed from each well and was serially diluted (1:10 dilution) in sterile phosphate buffer solution (PBS). The diluted suspension (20 μL) was subsequently removed and plated on Muller-Hinton agar (MHA) in duplicate for bacterial counting after incubation for 18–21 h at 37°C.

### Evaluation of the Mechanism of Action

#### SYTOX Green Uptake Assay

SYTOX green is a membrane-impermeable dye that activates and fluoresces upon binding to the DNA. The assay was performed using a previously established method, with a slight modification. (Mayandi et al., 2020) Briefly, the bacteria were cultured overnight in MHB (20 μL) for 16–18 h. Subsequently, the bacterial suspension was vortexed, washed twice and suspended in sterile HEPES buffer solution (5 mM HEPES, 5 mM glucose, 7.4 pH) to obtain an OD_600_ of 0.3. An aliquot of 5 mM SYTOX green stock solution in DMSO was added to the bacterial suspension to obtain a final dye concentration of 2 μM. The mixture was incubated for 15 min at room temperature while being protected from light. The dye-loaded cell suspension (600 μL) was then added into a stirring quartz cuvette and inserted into a QuantaMaster spectrofluorometer for fluorescence time-based scan at 504 nm excitation and 523 nm emission. Once a constant fluorescence level was achieved, a concentrated peptide solution in water (1 μL) was added into the cuvette in order to obtain a desired final concentration of CaD23 (2x MIC) in the cell suspension. The change in fluorescence intensity was monitored until a stable range was observed. Maximum fluorescence was documented via the addition of Triton-X (final concentration of Triton-X 0.1% (v/v) in 600 μL cell suspension) into the cuvette. The fluorescence intensity (I) of the peptide-treated suspension was calculated and plotted as: (I_peptide_/I_Triton-X(max)_) x 100%

#### Molecular Dynamics Simulations

Established molecular dynamics (MD) simulations-based models were used to examine the interactions between the synthetic peptides and models of the bacterial and mammalian membranes, using the GROMACS 5.1 package. (Asbell et al., 2020) The ability of peptide to permeate or interact with the bacterial membrane and mammalian membrane served as a proxy for its antimicrobial efficacy and toxicity, respectively. The bacterial membrane was modelled using a mixture of phosphoethanolamine and phosphatidylglycerol lipids (3:1 ratio) whilst the mammalian membrane was modelled using phosphotidylcholine. Each membrane patch consists of 128 lipid molecules. The initial coordinates of each membrane patch were constructed using CHARMM-GUI, (Brooks et al., 2009; Lee et al., 2016) followed by 100 ns to equilibrate the system. Similar method had been successfully used by other research groups. (Richter et al., 2017; Domene et al., 2021)

The peptide was modelled using the AMBER14sb force field, and the lipid molecules were modelled using the AMBER lipid17 force field. Initially, the peptide, modelled in a helical conformation, was placed 4 nm above the membrane center, followed by solvation with water molecules using the TIP3 model of each system. (Jorgensen et al., 1983) Counter ions were added to neutralise each system. Each system was first subjected to 500 steps of energy minimisation, followed by 20 ps of MD simulation in the canonical NVT ensemble (N = constant number; V = volume; T = temperature). Each system was first simulated for 400 ns to allow the peptide to adsorb on the membrane surface. Due to the complex free energy landscape of the peptide-membrane system, the time scale required to reach the equilibrium state was considerably lengthy. To overcome this difficulty, 400 ns simulations of simulated annealing, as outlined by Farrotti et al., (Farrotti et al., 2015) were performed. In each simulated annealing cycle, the temperature of the system was increased from 300 to 375 K in 50 ps steps, followed by a 1 ns simulation at 300 K. This was followed by 400 ns of normal MD simulation at 300 K. To understand the conformation of CaD23 in water, we carried out 100 ns of Hamiltonian replica exchange MD (HREMD) simulations, (Bussi, 2014) starting from a random structure. The LINCS algorithm (Hess et al., 1998) was applied to restrain the bond between hydrogen atoms and heavy atoms, enabling a time step of 2 fs Both Lennard-Jones and short-range electrostatic interactions were set to extend to 0.9 nm, while the long range electrostatic interactions were calculated using particle mesh Ewald method. (Essmann et al., 1995) The temperature and pressure were controlled by Nose–Hoover (Nosé and Klein, 1983) and semi-isotropic Parrinello–Rahman algorithms, (Martonák et al., 2003) respectively. Pairwise atom-positional root mean-square deviation (RMSD), based on Kabsch’s formula, was also calculated to evaluate the structural changes and heterogeneity of the CaD23 in water and in mimetic membranes. (Brüschweiler, 2003) A higher distribution of pairwise RMSD values would suggest an intrinsic flexibility of the peptide.

### Circular Dichroism (CD) Spectroscopy

Circular dichroism (CD) spectroscopy was performed using Chirascan CD Spectrometer (Applied Photophysics, Surrey, United Kingdom) to examine the secondary structures/conformations of CaD23 in water and in 30% trifluoroethanol (TFE), adapted from previous established protocols. (Fort and Spray, 2009; Mayandi et al., 2020) 30% TFE has been shown to probe and stabilise secondary structures such as helical folding (if present), which corresponds well with the structural findings obtained from NMR spectroscopy. (Sönnichsen et al., 1992; Howard and Smales, 2005)

Briefly, CaD23 was prepared and dissolved in the respective solutions, with a final concentration of 500 μg/ml. Far UV-CD spectra of the peptides were obtained via a Chirascan CD Spectrometer using a 0.1 cm path length quartz cuvette at 20°C. The spectra were obtained between 190 and 260 nm at a step size of 1.0 nm, and the final spectrum was taken to be the average of four scans. The CD spectra were subsequently subtracted from the background (buffer without CaD23) and were analysed.

## Results

### Peptide-antibiotic Interaction

#### Checkerboard Assay

The MICs of CaD23, amikacin and levofloxacin against Gram-positive and Gram-negative bacteria are presented in Table 1. A number of peptide-antibiotic combinations were examined for their interactive antimicrobial effect against both Gram-positive and Gram-negative bacteria (Table 2). It was found that both CaD23-amikacin combination demonstrated a strong additive effect against SH1000 (FICI = 0.60 ± 0.04) and MRSA43300 (FICI = 0.56 ± 0.19). Similarly, CaD23-levofloxacin combinations achieved strong additive effects against SH1000 (FICI = 0.69 ± 0.11) and MRSA43300 (FICI = 0.60 ± 0.13). On the other hand, CaD23-amikacin and CaD23-levofloxacin combinations demonstrated an indifferent effect against PA19660 (FICI = 1.08–1.15) and PA27853 (FICI = 1.03–1.04).

**TABLE 1:** Minimum inhibitory concentration (MIC) of CaD23, amikacin and levofloxacin against methicillin-sensitive *Staphylococcus aureus* (SH1000), methicillin-resistant *S. aureus* (ATCC MRSA43300), *Pseudomonas aeruginosa* ATCC PA19660 (cytotoxic strain), ATCC PA27853 (invasive strain) and PAO1L (invasive strain). The MIC value is expressed in μg/ml (and μM in bracket).

Treatment	SH1000	MRSA43300	PA19660	PA27853	PAO1L
CaD23	12.5 (5.2)	25 (10.4)	25 (10.4)	25 (10.4)	50 (20.8)
Amikacin	1.25 (2.1)	2.5 (4.3)	0.63 (1.1)	1.25 (2.1)	0.63 (1.1)
Levofloxacin	0.31 (0.86)	0.31 (0.86)	0.31 (0.86)	0.63 (1.7)	0.31 (0.86)

All assays were conducted as three independent experiments in biological duplicate. The presented values are the mean values. Standard deviation is not presented herein as the same results are consistently observed in all three independent experiments.

**TABLE 2:** Evaluation of the interactive antimicrobial effect of CaD23 and antibiotics, including amikacin and levofloxacin, using checkerboard assay. Experiments were conducted against methicillin-sensitive *Staphylococcus aureus* (SH1000), methicillin-resistant *S. aureus* (ATCC MRSA43300), and *Pseudomonas aeruginosa* ATCC PA19660 (cytotoxic strain) and ATCC PA27853 (invasive strain).

Treatment	Bacteria	FICI^a^	Interpretation
CaD23 + Amikacin	SH1000	0.60 ± 0.04	Additive
	MRSA43300	0.56 ± 0.19	Additive
	PA19660	1.15 ± 0.10	Indifferent
	PA27853	1.04 ± 0.04	Indifferent
CaD23 + Levofloxacin	SH1000	0.69 ± 0.11	Additive
	MRSA43300	0.60 ± 0.13	Additive
	PA19660	1.08 ± 0.04	Indifferent
	PA27853	1.03 ± 0.03	Indifferent

MIC = Minimum inhibitory concentration; FICI = Fractional inhibitory concentration index.

a FICI is calculated as: (MIC_CaD23(combined)_/MIC_CaD23(alone)_) + (MIC_ami(combined)_/MIC_ami(alone)_).

FICI < 0.5 = Synergistic; FICI between 0.5–1.0 = additive; FICI >1 to 4 = indifferent; FICI >4 = antagonistic.

The results are based on three independent experiments performed in biological duplicate. The results are presented in mean ± standard deviation.

#### Time-Kill Kinetics Assay

**FIGURE 1:**
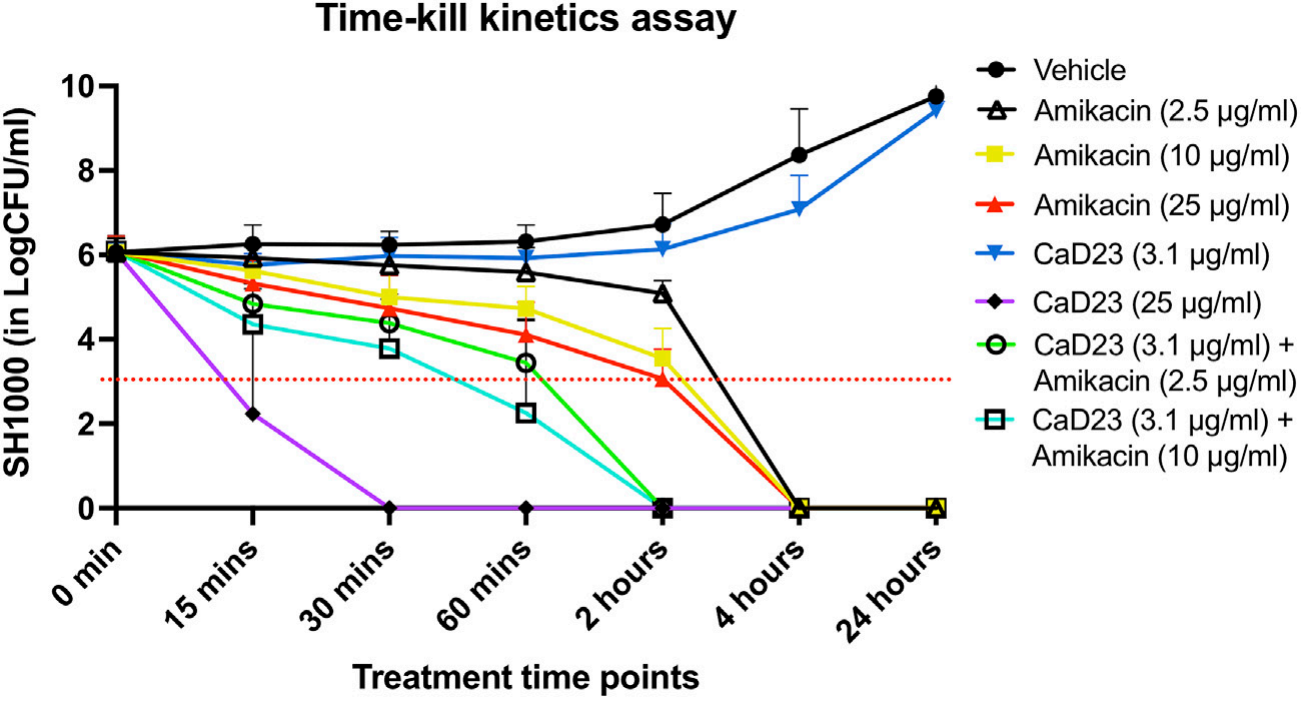
Time-kill kinetics assay examining the time- and concentration-dependent anti-bacterial effect of CaD23 (0.25x MIC and 2x MIC), amikacin (2x, 8x, and 20x MIC) and combined CaD23-amikacin against *S. aureus* (SH1000) over 24 h. The MIC of CaD23 and amikacin against SH1000 was 12.5 μg/ml and 1.25 μg/ml. SH1000 incubated with phosphate buffer solution (PBS) serves as the untreated control/vehicle. “0 min” represents the starting inoculum, which is around 6 log_10_ CFU/ml. The red dotted horizontal line at 3 log_10_ CFU/ml signifies the threshold of significant bacterial killing (defined as 99.9% or 3 log_10_ CFU/ml reduction of the bacterial viability compared to the starting inoculum). Data is presented as mean ± standard deviation (depicted in error bars) of three independent experiments performed in biological duplicate.

Based on the results of the checkerboard assay, the concentration- and time-dependent antimicrobial effect of combined CaD23-amikacin against SH1000 was further explored. The MIC of CaD23 and amikacin against SH1000 was 12.5 μg/ml and 1.25 μg/ml, respectively. When CaD23 was used alone at the concentration of 25 μg/ml (2x MIC), it was able to achieve 99.9 and 100% killing of SH1000 by 15 and 30 min post-treatment, respectively (Figure 1). This was significantly faster than amikacin at 2.5 μg/ml (2x MIC), 10 μg/ml (8x MIC) or 25 μg/ml (20x MIC), which only achieved 100% killing of SH1000 by 4 h post-treatment (i.e. 8 times slower). The addition of CaD23 at sub-MIC level (3.1 μg/ml; 0.25x MIC) expedited the antimicrobial action of amikacin (2.5 μg/ml; 2x MIC) and amikacin (10 μg/ml) against SH1000 by 2–4 times (for 99.9% killing) and 2 times (for 100% killing) when compared to amikacin (2.5 μg/ml) and amikacin (10 μg/ml) alone, respectively. This also suggests that combination treatment enables more effective and efficient killing of bacteria at lower treatment concentrations of CaD23, which serves as a useful strategy to reduce the concentration-dependent toxicity of CaD23.

### Mechanism of Action of CaD23

#### SYTOX Green Uptake Assay

**FIGURE 2:**
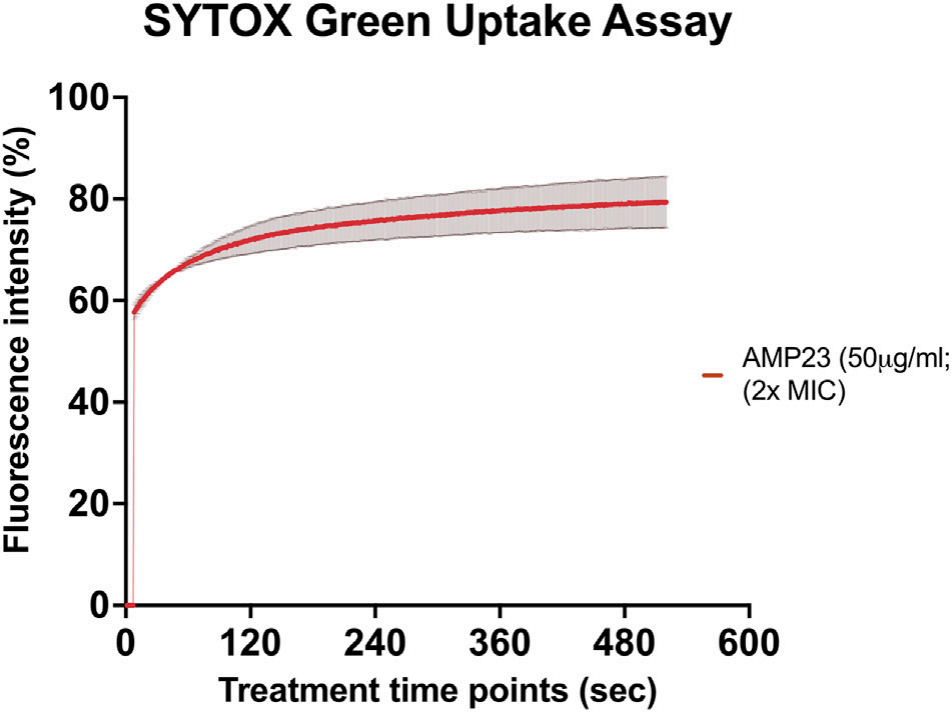
Membrane permeabilising action of CaD23 against *S. aureus* ATCC SA29213 determined by SYTOX green uptake assay. The graph demonstrating rapid membrane permeabilising action of CaD23 (50 μg/ml; 2x MIC) against SA29123, with a 60% increase in fluorescence intensity (due to SYTOX green uptake) within seconds of treatment and plateaued at ∼80% fluorescence intensity at 8 min. The fluorescence intensity is presented as mean ± standard deviation (depicted in error bars) of two independent experiments. The maximum fluorescence intensity (100%) was derived from the positive control, Triton-X 0.1% (v/v). Fluorescence intensity (I) of the peptide-treated suspension was calculated and plotted as: (I_peptide_/I_Triton-X(max)_) × 100%. The study was conducted as three independent experiments.

SYTOX green uptake assay was performed to study the underlying mechanism of action of CaD23 against *S. aureus* ATCC SA29213 (MIC = 25 μg/ml). It was shown that CaD23 at 50 μg/ml (2x MIC) exhibited rapid membrane permeabilisation of SA29213, with 60% SYTOX green uptake observed within seconds of treatment and reaching 80% membrane permeabilisation at around 8 min post-treatment (Figure 2).

#### Molecular Dynamics (MD) Simulations

**FIGURE 3:**
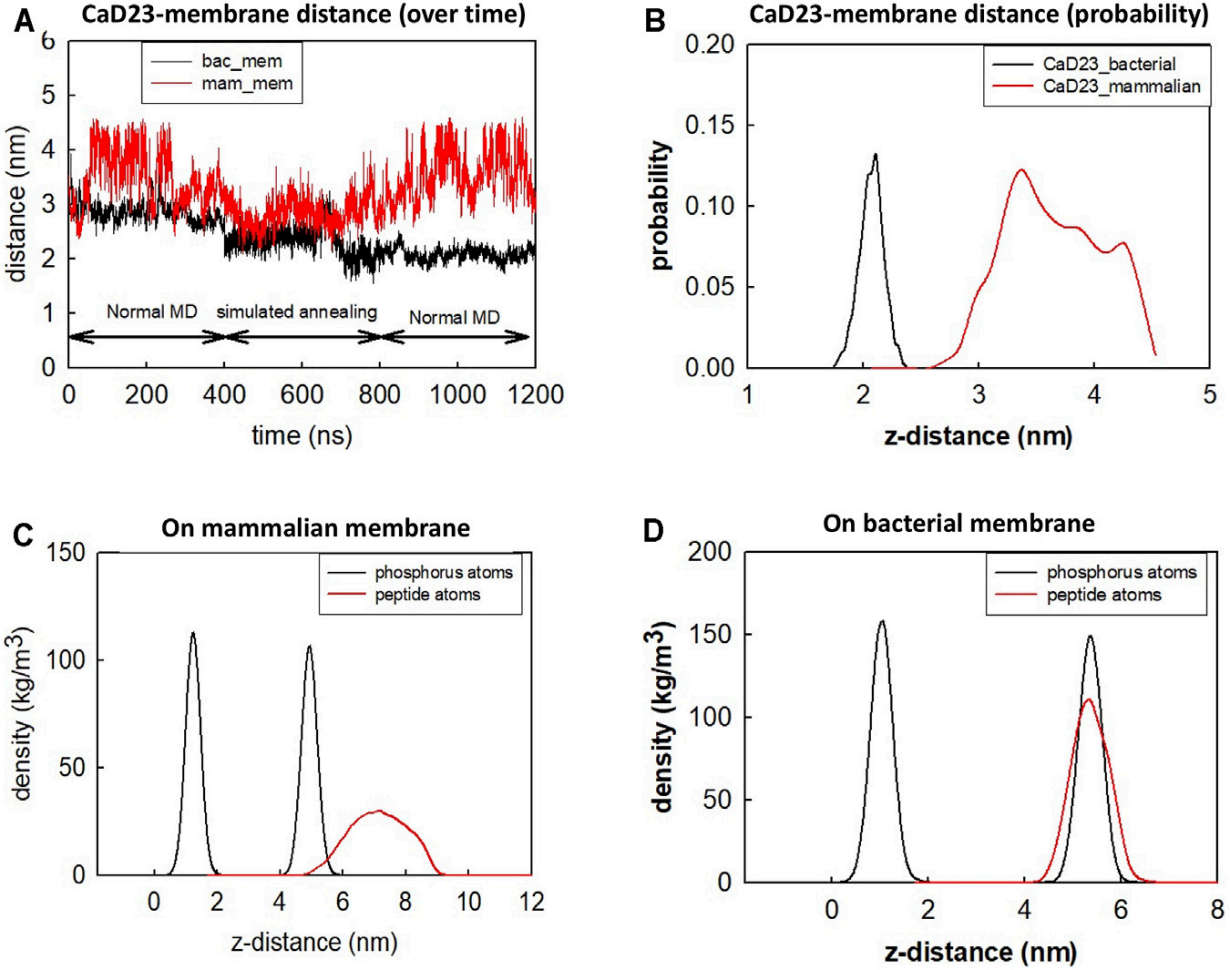
Molecular dynamics simulation of CaD23 on model mammalian and bacterial membranes. Each simulation was run for 400 ns at 300 K, followed by another 400 ns using simulated annealing (SA) to accelerate phase space sampling, finally followed by a further 400 ns simulation to obtain equilibration. **(A)** The graph showing the distance between CaD23 and mammalian or bacterial membrane over 1,200 ns CaD23 is shown to be closer to the bacterial membrane than to the mammalian membrane, suggesting a stronger interaction between CaD23 and the bacterial membrane. **(B)** The probability distribution of the peptide-membrane distance in the last 400 ns, demonstrating a closer distance of CaD23 to the bacterial membrane than to the mammalian membrane. **(C-D)** Density distributions of the CaD23 with respect to the phosphate groups of the bilayer membranes. The analysis is based on the last 400 ns simulation.

**FIGURE 4:**
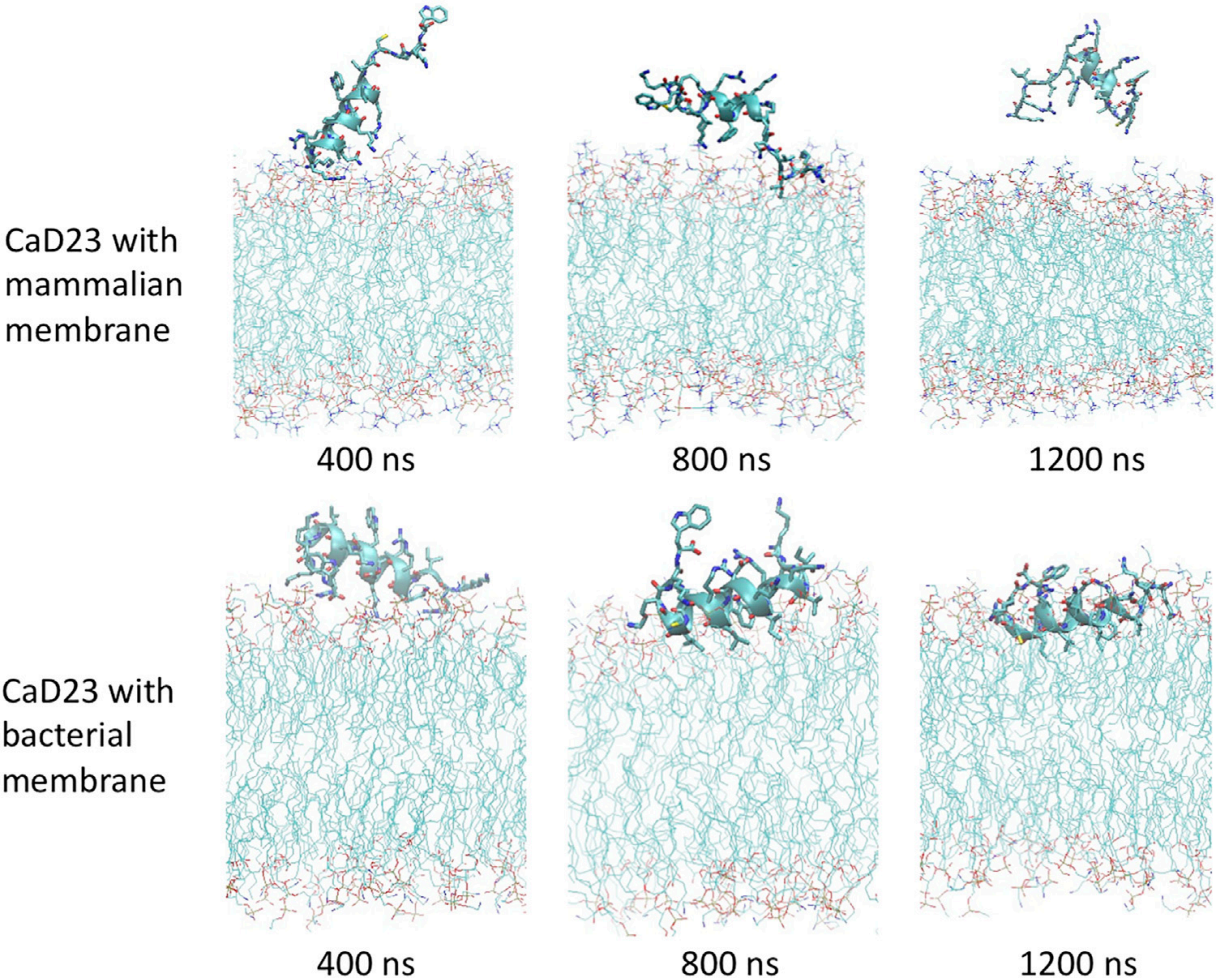
Molecular dynamics simulations study visualising the interaction between CaD23 and mammalian/bacterial membranes at an atomistic level. Representative snapshots of CaD23 with mammalian and bacterial membranes. The conformation of each snapshot corresponds to the most common configuration of CaD23 during the last 400 ns simulations. The snapshots demonstrate a stronger interaction (a closer distance) between CaD23 and bacterial membrane than mammalian membrane, corresponding with the experimental data on efficacy (high) and toxicity (low). This also suggests that the rapid action of CaD23 is likely attributed to its membrane-permeabilising action.

To understand the mode of interactions of the CaD23 peptide with the membranes, MD simulations of CaD23 with model bacterial and mammalian membranes were carried out. The distance between the centre of mass of CaD23 and the bilayer centre of mammalian and bacterial membranes is shown in Figures 3A,B. In the first 400 ns, the distance between CaD23 and both membranes decreased, suggesting a rapid adsorption of CaD23 on both membranes. CaD23 was closer to the bacterial membrane (z-distance = 2 nm) than the mammalian membrane (z-distance = 3.5 nm with considerable fluctuation), suggesting a stronger peptide-bacterial membrane interaction. Representative snapshots of the MD simulations of CaD23 with mammalian and bacterial membranes are shown in Figure 4.

Upon adsorption on the membrane, CaD23 started to interact with the head groups of the membrane, which involved the rearrangement of the head groups and the penetration of hydrophobic residues of CaD23 into the membrane (Figure 4). Due to complex mode of interactions, this process was characterized by a frustrated free energy landscape. To accelerate sampling, simulated annealing (SA) was applied. The peptide-membrane distance was found to decrease further, particularly for the distance between CaD23 and the bacterial membrane, because the strong perturbation of the bacterial head groups facilitated the penetration of the hydrophobic residues of CaD23 into the lipid tail region of the bacterial membrane, which did not occur on the mammalian membrane due to weak interactions. To obtain an equilibrium state, classical MD simulations without SA were carried out for a further 400 ns The distance between the peptide and the bacterial membrane decreased further and remained stable. In contrast, the distance between CaD23 and the mammalian membrane increased and fluctuated with many adsorption-desorption events on the mammalian membrane, suggesting a weaker interaction.

The different locations of CaD23 with respect to the bilayer center can also be seen from the density distribution of CaD23 with respect to the phosphate atoms during the final 400 ns of the MD simulations (Figures 3C,D). On the mammalian membrane, the peak of CaD23 was low and the distribution of CaD23 was wide and far away from the phosphate groups, suggesting a low affinity of CaD23 to the mammalian membrane. In contrast, the peak of CaD23 was close to the phosphate groups upon strong adsorption on the bacterial membrane.

**FIGURE 5:**
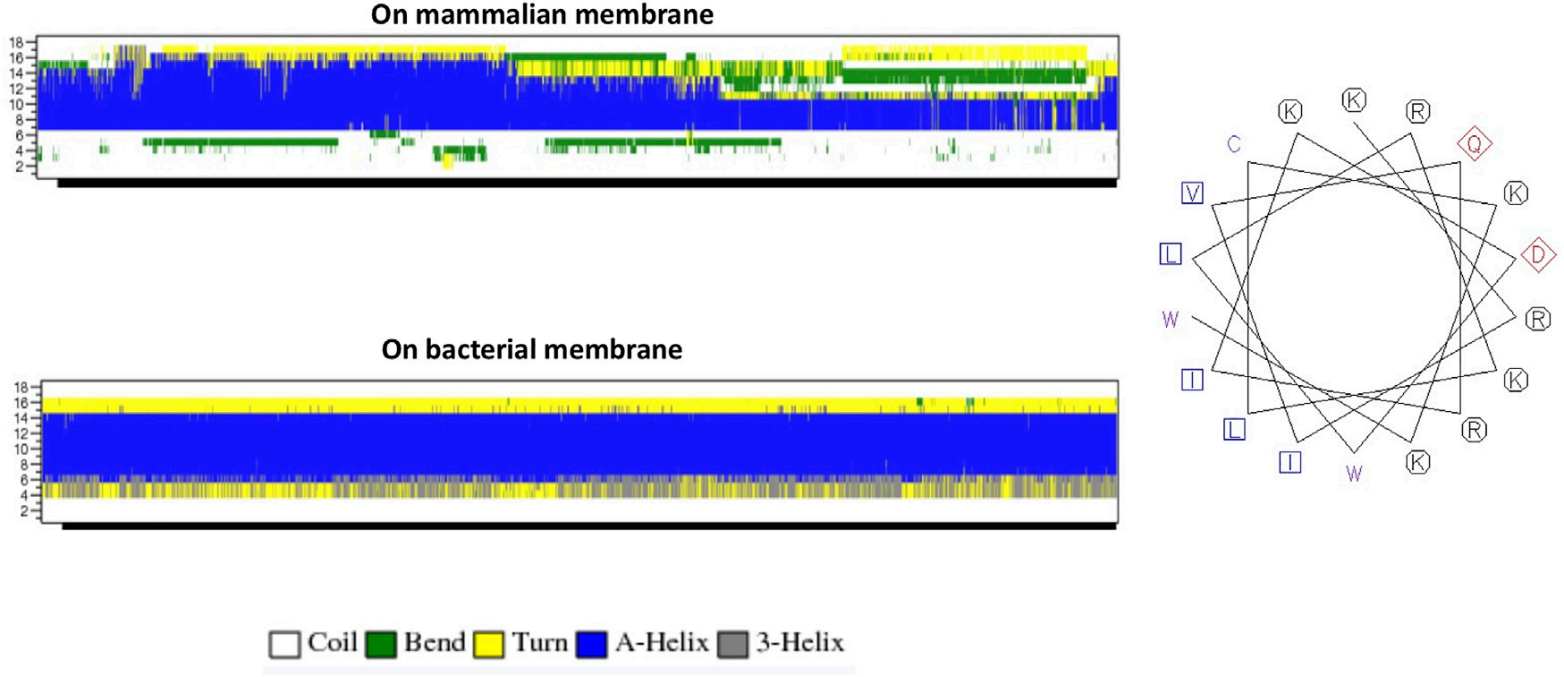
**(A)** Secondary structure evolution of CaD23 during the molecular dynamics (MD) simulations. CaD23 adopts a partially alpha-helical structure on the mammalian membrane compared to a highly alpha-helical structure on the bacterial membrane. **(B)** The helical wheel plot of CaD23. Blue and purple letters represent hydrophobic residues, red letters represent negatively charged acidic residues, and black letters represent positive charged basic residues.

**FIGURE 6:**
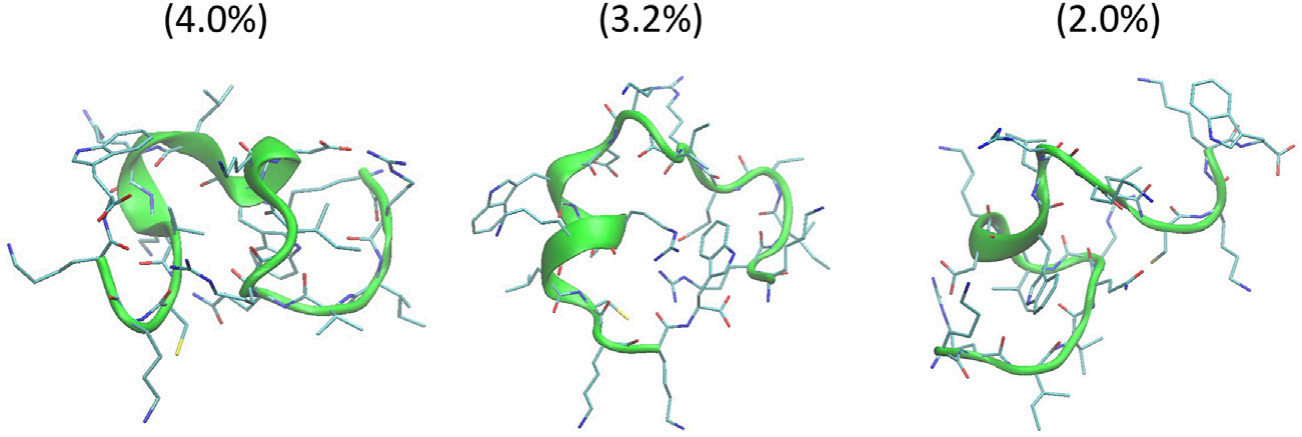
Conformations of the top three clusters of CaD23 in solution, calculated from the last 50 ns of the total 100 ns of Hamiltonian replica exchange molecular dynamics (HREMD) simulations. The numbers in the bracket were the population of each cluster.

**FIGURE 7:**
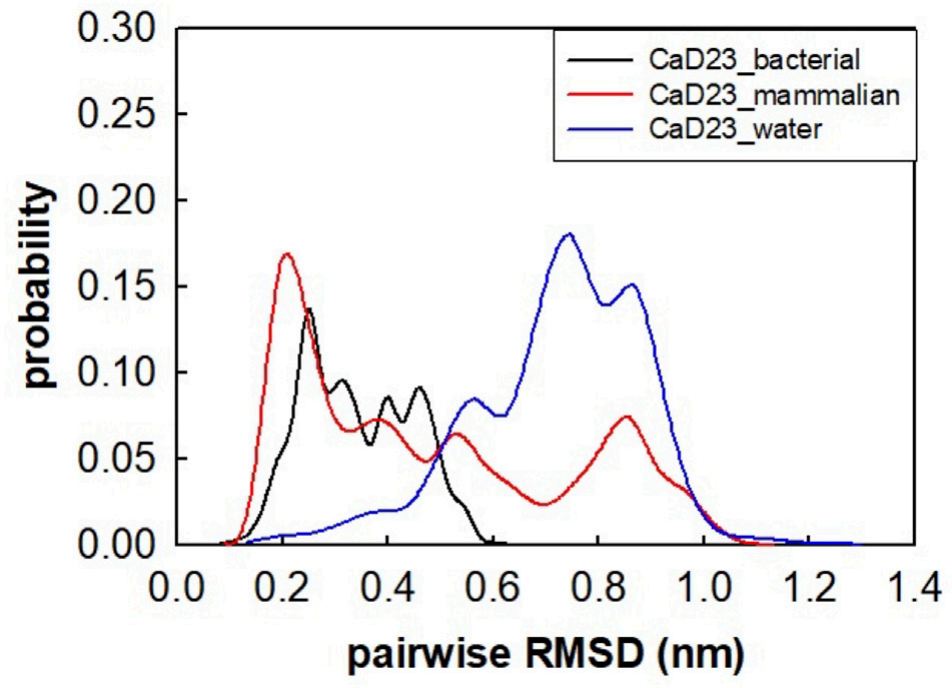
Pairwise root mean-square deviation (RMSD) distribution of CaD23 in water and in mimetic bacterial and mammalian membranes. The RMSD values of CaD23 in water were high and widely distributed, suggesting that the conformation was highly flexible and heterogeneous in water when compared to in mimetic membranes.

**FIGURE 8:**
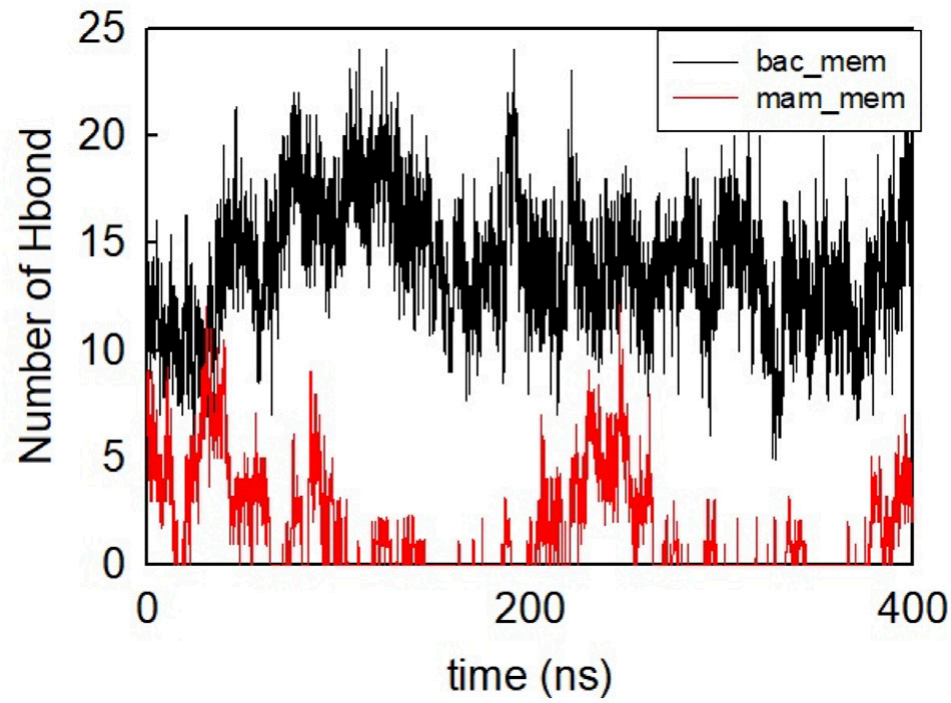
Number of hydrogen bonds formed between CaD23 and the two membranes during the last 400 ns

The helical wheel revealed that when CaD23 was in helical conformation, it formed a perfect facial amphiphilic conformation, with positively charged residues facing one side and the hydrophobic residues facing the other side (Figure 5). Although CaD23 largely maintained the helical conformation on both membranes, it was more helical on the bacterial membrane than on the mammalian membrane. On the other hand, the conformation of CaD23 in water was shown to be highly flexible with no distinct secondary structure (Figure 6). The structural flexibility and heterogeneity were further demonstrated by the large and widely distributed pairwise RMSD in water, compared to in mimetic membranes (Figure 7). The snapshots from the last 400 ns in Figure 4 clearly demonstrate that the peptide adopts a helical conformation on the bacterial membrane, with the hydrophobic residues inserted into the lipid tail region while the basic residues interact with the head groups, resulting in perturbation of the membrane-water interface. On the mammalian membrane, CaD23 was only partially helical and fluctuated due to the lack of strong electrostatic interactions, resulting in less perturbation of the mammalian membrane. Moreover, CaD23 formed more hydrogen bonds with the bacterial membrane compared to the mammalian membrane (Figure 8), which further contributed to the high affinity to the bacterial membrane.

### Secondary Structures of Peptide

**FIGURE 9:**
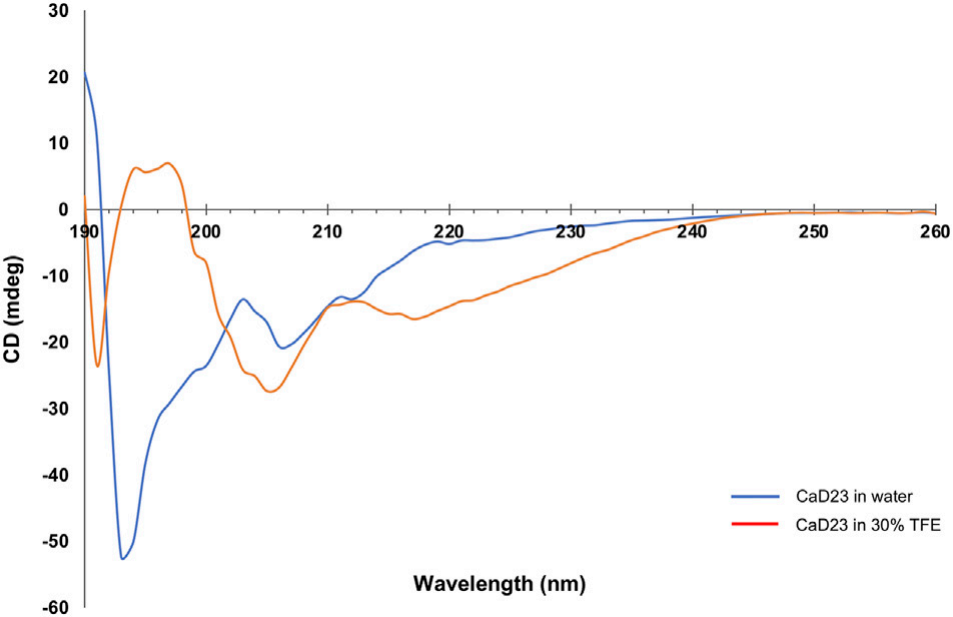
Secondary structures of CaD23 in water and in 30% trifluoroethanol (TFE) determined by circular dichroism (CD) spectroscopy. CaD23 was shown to adopt a random coil conformation in water and an alpha-helical conformation in 30% TFE, corroborating the findings of molecular dynamics simulations.

CD spectroscopy was used to examine the secondary structures of CaD23 in water and in 30% TFE. In water, CaD23 was shown to be in a random coil conformation, with a peak negative band at 194 nm and a lack of typical alpha-helical or beta-sheet spectrometric findings (Figure 9). (Greenfield, 2006) In 30% TFE, CaD23 adopted a highly alpha-helical conformation, with a positive band at 194 nm and double negative bands at 206 and 218 nm, which was very similar to the readings of a classical alpha-helical conformation. (Greenfield, 2006) These findings corresponded well with the findings of MD simulations, which demonstrated CaD23 in a random coil conformation in water and an alpha-helical conformation in the vicinity of both bacterial and mammalian mimetic membranes (with a stronger alpha-helical folding with bacterial membrane).

## Discussion

The serendipitous discovery of HDPs in the early 1980s has sparked a significant interest in the field of antimicrobial therapy as HDPs have been shown to exhibit broad-spectrum and rapid antimicrobial action, with low risk of developing AMR. However, a number of barriers, including toxicity to host cells/tissues, have so far impeded the translation of HDP-based treatment to clinical use. In this study, we demonstrated that CaD23 could enhance the antimicrobial efficacy of commonly used antibiotics, including amikacin and levofloxacin, in a strong additive manner, against methicillin-sensitive and methicillin-resistant *S. aureus* when they were used in combination. This suggests that a lower treatment concentration of CaD23 and antibiotic can be used, serving as a useful strategy to reduce the concentration-dependent drug toxicity that is often observed in clinical practice. (Forster et al., 2005; Dua et al., 2012)

Furthermore, the addition of CaD23 at sub-MIC level was able to expedite the antimicrobial action of amikacin by 2–4 times when used in combination. Theoretically, such beneficial effect can reduce the risk of developing AMR as the bacteria have less time to adapt and develop effective mechanisms against the antibiotics. Studies have shown that bacteria treated by membrane-active peptides with rapid antimicrobial action have a low risk of developing AMR whereas bacteria treated by conventional antibiotics are prone to developing AMR, especially when they are chronically used at a sub-MIC level. (Hancock and Sahl, 2006; Llor and Bjerrum, 2014; Mayandi et al., 2020; Ting et al., 2021d) This is due to the fact that modification of the entire membrane of the microorganisms in response to membrane-active peptides incurs a high fitness cost when compared to alteration of a particular binding site targeted by conventional antibiotics (e.g. alteration in the penicillin-binding protein reduces the efficacy of beta-lactam antibiotics). (Kapoor et al., 2017; Ting et al., 2020b)

The advantageous strong additive effects of CaD23-amikacin and CaD23-levofloxacin against Gram-positive bacteria are likely attributed the different underlying mechanism of action of these drugs. Amikacin is a commonly used aminoglycoside in clinical practice (including ophthalmology) that exhibits its antimicrobial activity via inhibition of the 30S ribosomal subunit, (Krause et al., 2016; Ting et al., 2021b) whereas levofloxacin, a frequently used fluoroquinolone, kills bacteria by inhibiting the bacterial DNA gyrase. It is likely that CaD23 interacts and permeabilises the cytoplasmic membrane of Gram-positive bacteria and facilitates the penetration of aminoglycoside and levofloxacin into the bacterial cells, enabling a more effective binding to the intracellular targets.

Interestingly, we did not observe the same antimicrobial additive effect when CaD23 was used in combination with either amikacin or levofloxacin against Gram-negative bacteria. One of the main differences between Gram-positive and Gram-negative bacteria lies in the different compositions of the bacterial cell envelope. (Silhavy et al., 2010) While both types of bacteria have a cytoplasmic/inner membrane, Gram-positive bacteria possess a thick peptidoglycan outer layer whereas Gram-negative bacteria possess an additional outer membrane, which is primarily composed of negatively charged lipopolysaccharides (in the outer leaflet of the outer membrane). (Kapoor et al., 2017) It is likely that CaD23 primarily acts on the inner cell membrane (of both types of bacteria), with a weaker interaction with lipopolysaccharides, thereby explaining the additive effects of combined CaD23-antibiotic that were observed in Gram-positive bacteria but not in Gram-negative bacteria. Further investigations are warranted to understand the lack of mode of activity of CaD23-antibiotic combination against Gram-negative bacteria.

On the other hand, our group had recently demonstrated that FK16 (a truncated version of LL-37) was able to enhance the antimicrobial activity of vancomycin against *P. aeruginosa*. (Mohammed et al., 2019) Vancomycin is a glycopeptide antibiotic that has poor permeability against the outer membrane of Gram-negative bacteria. (Antonoplis et al., 2019) It was hypothesised that FK16, a membrane-active peptide, permeabilizes the outer membrane of the Gram-negative bacteria and improves the delivery of vancomycin to access periplasmic cell wall precursors and intracellular target. Antonoplis et al. (2019) had similarly demonstrated the synergistic effect in a vancomycin-arginine peptide conjugate in treating carbapenem-resistant *Escherichia coli*, likely through a similar mechanism of action described above. Kampshoff et al. (2019) observed a synergistic effect between ciprofloxacin and melimine (a highly cationic, hybridised peptide derived from melittin and protamine) against ciprofloxacin-resistant *P. aeruginosa*, but not against *S. aureus* or non-drug resistant *P. aeruginosa*. In addition, a synergistic effect was not observed in either melimine-cefepime (a fourth-generation cephalosporin), Mel4 (truncated melimine)-ciprofloxacin, or Mel4-cefepim, highlighting the heterogeneous interactions among different types of peptides and antibiotics.

Both SYTOX green uptake assay and MD simulation studies demonstrated that CaD23 achieved its antimicrobial activity *via* a membrane-permeabilising action. In the recent decades, MD simulations have been increasingly utilised in the process of drug discovery and development in many fields, including the field of HDPs. (Durrant and McCammon, 2011; Li et al., 2012; De Vivo et al., 2016; Li et al., 2017; Ting et al., 2020b) They have been shown to predict the secondary structures of proteins/peptides, decipher the underlying mechanism of action, and identifying key residues responsible for the protein-protein or protein-membrane interaction at an atomistic level. (Tsai et al., 2009; Li et al., 2017; Li et al., 2018) As the chemical space of synthetic and natural HDPs is vast, MD simulation serves as a powerful tool to expedite the process of designing and optimising the peptide sequences as it reduces the need for repetitive microbiological assays and laborious screening of a large amount of peptide that is usually required in traditional mutation-based empirical methods.

A number of key factors, including alpha-helicity, amphiphilicity, cationicity and hydrophobicity, have been described to influence the antimicrobial efficacy of HDPs. (Hancock and Sahl, 2006; Ting et al., 2020b; Mookherjee et al., 2020) In our study, MD simulations have revealed a number of important findings pertaining to the CaD23 molecule. Firstly, we observed a rapid adsorption of CaD23 on the negatively charged bacterial membrane during the early stage of the simulation (particularly at the N-terminus where the Lys1 is located), highlighting the importance of cationicity in the CaD23 molecule. In contrast, the zwitterionic nature of the mammalian membrane exhibited a weaker interaction with CaD23. Secondly, we showed that CaD23 adopted a more alpha-helical conformation on the bacterial membrane than the mammalian membrane, suggesting that alpha-helicity plays an important contributory role to the antimicrobial efficacy of CaD23. In the helical conformation, the peptide displays high facial amphiphilicity, which resulted in a more favourable interaction with the bacterial membrane, with a deeper penetration of CaD23 into the bacterial membrane. This is in accordance with many studies in the literature that had highlighted the important correlation between alpha-helicity and antimicrobial efficacy observed in various natural and synthetic HDPs. (Haney et al., 2019; Mookherjee et al., 2020) We also observed that the Trp18 residue at the C-terminal had a strong interaction with the bacterial membrane but not the Trp10 residue. This suggests that the Trp10 residue may potentially be substituted with a less hydrophobic residue such as Leu or Ile to reduce the hydrophobicity and toxicity, and to improve its water solubility. Experimental data from CD spectroscopy further validated the secondary structures of CaD23 (i.e. a random coil conformation in water and an alpha-helical conformation in 30% TFE), highlighting the value of MD simulations in predicting the secondary structures of peptides.

Despite the many advantages of MD simulations described above, it is noteworthy to mention that the model bacterial membrane utilised in the current MD simulation is only representative of the inner membrane of the Gram-positive and Gram-negative bacteria. Atomistic models have been developed for bacterial outer membrane and several studies have been carried out to understand the structural dynamics of the outer membrane. (Pontes et al., 2012; Li et al., 2020a; Li et al., 2020b) However, MD simulations with outer membrane is out of the scope of this study as CaD23 was mainly efficacious against Gram-positive bacteria. Another issue with conventional MD is that it often suffers from insufficient sampling and therefore requires multiple replicates. It has been shown that increasing the temperature can greatly accelerate conformational sampling, (Wang et al., 2016; Chen et al., 2019) hence we employed the simulated annealing method in our work. The simulated annealing lasted for 400 ns, which included multiple cycles of annealing, thus enabling the system to escape from being trapped in local minima. After the 400 ns simulated annealing, we carried out another 400 ns simulations to further equilibrate the system.

In summary, our study demonstrated that CaD23 is a membrane-active peptide that has the ability to enhance the antimicrobial action of commonly used antibiotics such as amikacin and levofloxacin, potentially offering a new therapeutic strategy for Gram-positive bacterial infection. Further *in vivo* studies to validate these results would be invaluable. MD simulation serves as a useful computational tool in deciphering the underlying mechanism of action and guiding the design process of HDPs. In addition, potential strategies, including rational residue substitution, N- and C-terminal modifications, introduction of unnatural amino acids, and nanoformulation, (Platania et al., 2019; Ting et al., 2020b; Casciaro et al., 2020; Puglia et al., 2020; Puglia et al., 2021) will be explored to further enhance the therapeutic potential of CaD23 in terms of antimicrobial efficacy and stability.

## Data Availability

The datasets presented in this study can be found in online repositories. The names of the repository/repositories and accession number(s) can be found in the article/Supplementary Material.
