# Supplementary figures and images for: Evaluation of Host Defense Peptide (CaD23)-Antibiotic Interaction and Mechanism of Action: Insights From Experimental and Molecular Dynamics Simulations Studies
Source: Front Pharmacol. 2021 Oct 7;12:731499. doi: 10.3389/fphar.2021.731499 (PMC8528955; doi:10.3389/fphar.2021.731499)

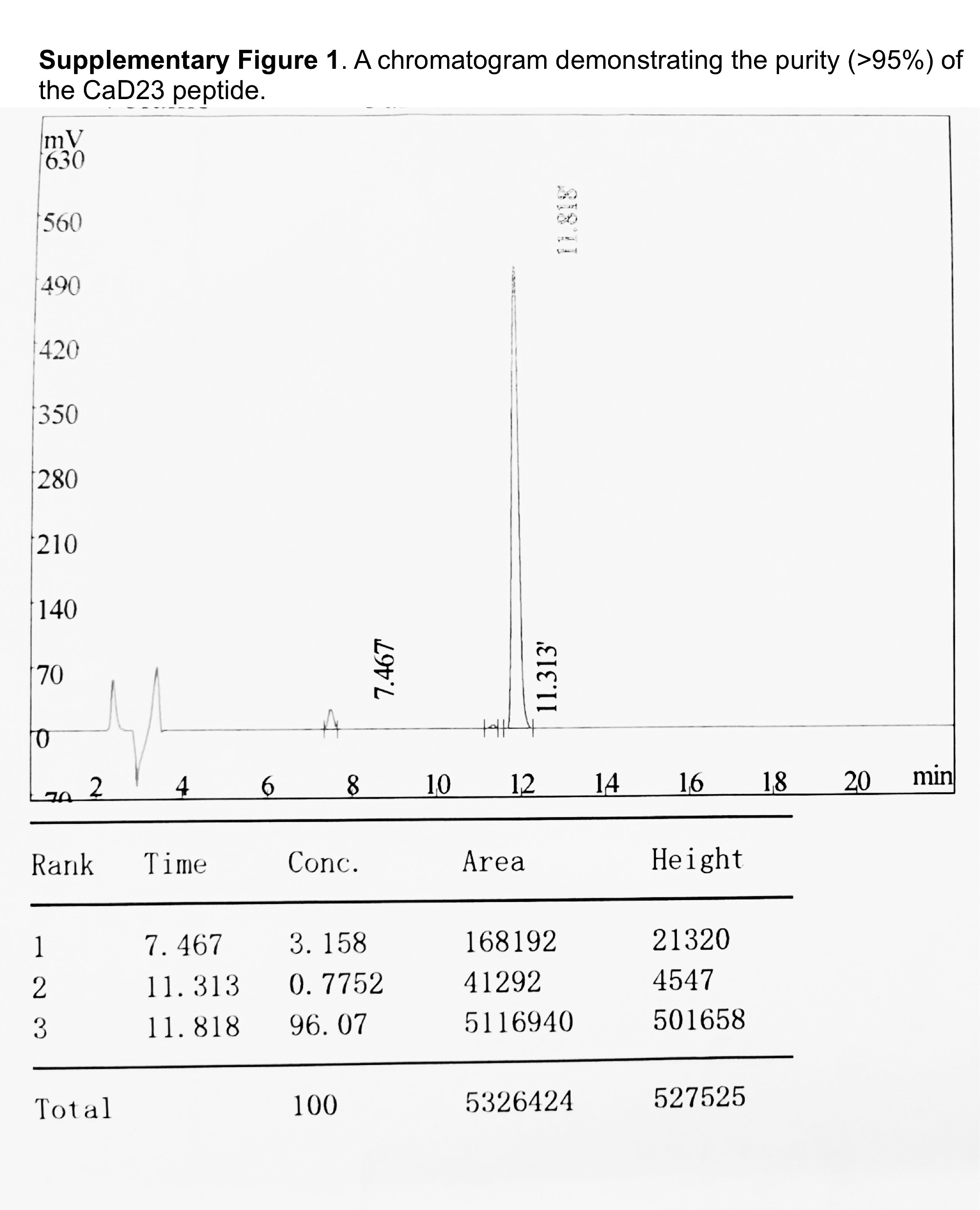

Supplement: Supplementary file 1 [file Image1.JPEG]
